# Full-Length Transcriptome Sequencing of *Pinus massoniana* Under Simulated *Monochamus alternatus* Feeding Highlights bHLH Transcription Factor Involved in Defense Response

**DOI:** 10.3390/plants14132038

**Published:** 2025-07-03

**Authors:** Quanmin Wen, Yajie Cui, Tian Xu, Yadi Deng, Dejun Hao, Ruixu Chen

**Affiliations:** 1 School of Landscape Architecture, Jiangsu Vocational College of Agriculture and Forestry, Zhenjiang 212499, China; 2 Co-Innovation Center for the Sustainable Forestry in Southern China, College of Forestry and Grassland, Nanjing Forestry University, Nanjing 210037, China; wqm@njfu.edu.cn (Q.W.); cuiyajie7@outlook.com (Y.C.); xutian@njfu.edu.cn (T.X.); dengyadi1@gmail.com (Y.D.)

**Keywords:** *Pinus massoniana*, *Monochamus alternatus*, transcription factor, transcriptome sequence

## Abstract

Background: *Pinus massoniana* is a significant lipid-producing tree species in China and a susceptible host for both the pine wood nematode and its insect vector, *Monochamus alternatus*. The basic helix–loop–helix (bHLH) family of transcription factors play a crucial role in responding to both biotic and abiotic stresses. However, the role of bHLH in terpene-induced defense in *P. massoniana* remains poorly studied. Results: Transcriptome sequencing using DNA Nanoball Sequencing (DNBSEQ) and PacBio Sequel platforms was performed, revealing differences in gene expression in *P*. *massoniana* branch under the simulated feeding treatment of methyl jasmonate (MeJA) spraying. Fifteen *bHLH* genes were cloned and analyzed, among which eight highly upregulated *PmbHLH* genes showed similar temporal expression after MeJA treatment and *M*. *alternatus* adult feeding. Five highly upregulated *bHLH* genes with nuclear localization were highly expressed in *P. massoniana* after *M. alternatus* feeding and interacted with the promoter of the terpene synthase gene *Pm TPS (−)-α-pinene*, confirming their involvement in the defense response of *P. massoniana* against the *M. alternatus* adult feeding. Conclusions: Our results unveil the temporal changes and the regulation of the induced defense system in *P. massoniana* mediated by both MeJA signaling and *M. alternatus* feeding treatment. The potential application for transgenic experiments and the breeding of resistant species in the future were discussed.

## 1. Introduction

Conifers integrate multiple constitutive and induced terpenoid-based defenses against natural enemies [1,2]. Inductive defenses target a regulatory mechanism that helps conifers cope with herbivore and pathogen attacks and involves the formation of traumatic resin ducts, secretion of induced oleoresin, selective expression of resin synthesis related genes, and enhanced activity of enzymes [3,4,5]. These oleoresins consist of volatile and nonvolatile terpenes, as well as phenolic compounds, which may attract natural predators, impact the mating and reproductive behaviors of certain herbivore species, and contribute to plant communication and nutrient exchange with the surrounding environment [6,7,8].

Over the past few decades, it has been discovered that several phytohormones, such as jasmonic acid (JA), ethylene (ET), abscisic acid (ABA), and salicylic acid (SA), are involved in the synthesis of chemical defenses and related signaling cascades [9,10,11]. MeJA is a stable exogenous derivative of JA that has been proven to be involved in plant growth regulation, stress responses, and secondary metabolism [12,13,14]. Due to its effectiveness, MeJA has been widely used to enhance the plant’s resistance to herbivorous insects for pest control in both agricultural and horticultural crops [15,16,17,18].

The jasmonates (JAs) are key components of the plant wound signal transduction cascade [19,20]. In the JA signaling pathway, JASMONATE ZIM-DOMAIN (JAZ) protein controls downstream MYC transcription factors, a subfamily of bHLH family that can recruit RNA polymerase and other transcription components to transcribe JA-response genes [21,22,23]. Current studies of bHLH transcription factors have shown that they typically act as direct or indirect activators of JA-associated defense genes [24,25]. There is substantial evidence that plant bHLH transcription factors regulate the expression of a large number of genes involved in plant responses to a wide range of plant growth and development stages, as well as responses to biological and abiotic stresses [26]. For example, the bHLH transcription factors modulate plant seed germination/dormancy [27,28], cell elongation [29], stomata development [30,31], flower initiation [32], and light-regulated development [33]. Meanwhile, a significant portion of the bHLH family is still considered to function as repressors of JA signaling [34,35], such as the bHLH transcription factors MYC2, MYC3, and MYC4, which jointly regulate jasmonate-mediated flowering inhibition in Arabidopsis [36]. In addition, bHLH transcription factors play vital roles in plant biosynthetic processes, such as amygdalin biosynthesis in almond (*Prunus dulcis* Miller (D. A. Webb), syn. *Prunus amygdalus* L.) [37], cyanogenic glucoside biosynthesis in *Lotus japonicus* induced by MeJA [38], anthocyanin biosynthesis [39,40], and terpenoid biosynthesis related to the resistance to insects and fungi [41]. The bHLH transcription factors also have been proven to be involved in in plant responses to abiotic stresses such as high temperature [42], drought stress [43], salt stress [44], and micronutrient deficiency stress [45]. In addition to the JA pathway, bHLH transcription factors are also involved in regulating the auxin (IAA) signaling pathway [46], salicylic acid (SA) signaling pathway [47], and abscisic acid (ABA) signaling pathway [48], as well as in the crosstalk between various plant hormone signaling pathways [49].

So far, most functionally characterized bHLH proteins are in Arabidopsis, and only a few *bHLH* genes have been functionally characterized in other plant species [50]. Conifers, the most widely distributed gymnosperms, are both ecologically and evolutionarily significant [51], yet our understanding of bHLH transcription factors in these species remains limited. Three putative *bHLH* genes encoding transcription factors homologous to *AtTT8* were identified in *Picea abies*, and yeast two-hybrid experiments suggested that the MYB-bHLH-WDR (MBW) complex may regulate the flavonoid pathway [52]. Similar studies in angiosperms have confirmed the role of the bHLH transcription factors in secondary metabolism [53,54,55,56]. A study of *P. sitchensis* based on microarray gene expression profiling suggested that bHLHs might be involved in defense responses triggered by mechanical injury or herbivore feeding in conifers [57]. However, strong evidence for their involvement in general stress responses, detoxification, redox regulation, or terpenoid or phenolic metabolism is still lacking. The bHLH transcription factors in conifers have also been hypothesized to participate in shade avoidance and tolerance [58], hypocotyl elongation [59], light and gibberellin signaling [60,61], brassinosteroid signaling [62], pine cone development [63], annual ring formation [64], paclitaxel biosynthesis [65], and resin production [66]. These findings highlight the potential roles of specific bHLH members in conifer growth and their responses to biotic and abiotic stresses.

The bHLH transcription factors recognize and bind to the E-box (CANNTG), the canonical G-box (CACGTG) of the target genes; meanwhile, their ability to dimerize or tetramerize allows them to interact with a broader range of cis-regulatory sequences [48]. These transcription factors typically contain a conserved bHLH domain of approximately 60 amino acids that consists of a basic region (b) at the N-terminal and a helix–loop–helix (HLH) region at the C-terminal [67]. Outside of the bHLH domain, however, the amino acid sequence across bHLH proteins shows limited conservation, which may be due to the presence of additional domains that help regulate their activity and DNA-binding specificity [26,68].

Pine wilt disease (PWD), caused by the pine wood nematode, is a highly destructive and internationally quarantined forest disease [69]. In Asia, *M. alternatus* is regarded as the most significant vector insect for the pine wood nematode [70]. Among affected countries, China faces the greatest threat. Since its first reported occurrence in Zhongshan Mausoleum, Nanjing, Jiangsu Province, in 1982 [71], the disease has rapidly spread to 16 provinces and 1 centrally administered municipality, affecting 616 counties and districts as of 2025 (National Forestry and Grassland Administration, Announcements No. 4 [2024] and No. 3 [2025]). PWD has resulted in widespread pine mortality and poses a serious risk to China’s 60 million hectares of pine forests. *P. massoniana* is one of the most widely distributed native afforestation species in southern China and provides essential materials for industry, agriculture, and medicine [72,73]. However, it is severely threatened by *M. alternatus* infestations and the pine wood nematode [74]. Early detection, removal of infected trees, and control of insect vectors are currently the most critical measures for managing PWD. However, in the long term, breeding and cultivating resistant native pine varieties represent a sustainable strategy for PWD prevention and control in China [69].

According to a previous study, resin terpenoid-induced defense plays a crucial role in protecting *P. massoniana* against *M. alternatus* and the pine wood nematode [75,76,77,78]. The treatments of exogenous MeJA treatment and *M. alternatus* adult feeding can induce the expression of terpene synthase genes to enhance resin terpenoid synthesis [79]. *α*-pinene is the most abundant monoterpene in *P. massoniana* resin, present both in the constitutive and induced resins, which acts as a strong attractive substance for *M. alternatus* [80,81]. The synthetase of *α*-pinene has been identified in *Pinus pinaster*, *Pinus pinea*, *Malvaceae Gossypium*, *Pinus elliottii*, and so forth, and its relative expression is significantly upregulated under biological stress [82,83,84]. For example, Liu et al. found that terpene synthase genes PmTPS4 and PmTPS21 were significantly upregulated in *P. massoniana* seedlings inoculated with pine nematode-resistant species, and the expression of the PmTSP4 protein reacted with GPP to synthesize *α*-pinene and a small amount of *β*-pinene and other terpenes [85]. All this evidence suggests that *α*-pinene is closely related to the induced defense of *P. massoniana*. However, due to limited genomic data and the lack of established genetic transformation methods, research on the upstream regulatory pathways of these functional genes remains unclear [86]. The response of bHLH transcription factors to MeJA signaling, along with their structure and function in *P. massoniana*, has not yet been reported. In this study, we identified five bHLH transcription factors in *P. massoniana* related to terpene synthase through transcriptome analysis following MeJA treatment to simulate *M. alternatus* adult feeding. This provides a theoretical foundation for understanding the regulation of terpene-induced defenses in the host plant, longhorn beetle and pine wilt nematode interaction, and the transmission mechanism of pine wilt disease.

## 2. Results

### 2.1. mRNA Sequencing and Assembly

**Figure 1:**
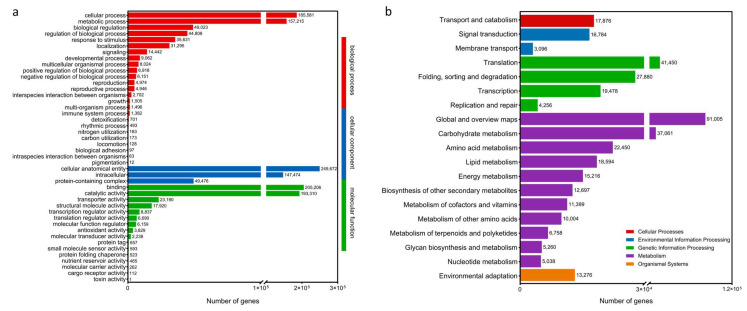
Annotation of transcripts in *P. massoniana.* Full-length transcriptomes from different databases. (**a**) GO classification of *P. massoniana* transcriptome isoforms. (**b**) KEGG pathway classification of *P. massoniana* transcriptome isoforms.

Full-length transcriptome data were obtained by the PacBio Sequel platform, and approximately 89.44 G subreads (998,540 circular consensus sequencing (CCS)) were generated, which have been uploaded to NCBI (PRJNA1205872). After filtering out incomplete CCS, 3,482,433 full-length nonchimeric read (FLNC) sequences with complete 5′–3′ ends were obtained. After clustering redundant sequences, 3,286,500 consensus sequences were obtained, and finally, 671,044 unigenes were obtained by clustering the same isoforms. The length of these sequences ranged from 198 to 21,157 bp. In this study, 671,044 unigenes were annotated using seven databases. The number of annotated unigenes in the seven databases ranged from 378,461 (56.40%, Swissprot) to 523,546 (78.02%, NR), and 585,792 (87.30%) unigenes were annotated in at least one database (Table S1). In addition, 21,697 unigenes were annotated to the GO database (Figure 1a), and 12,556 unigenes were annotated to the KEGG database (Figure 1b). A large number of genes were annotated using KOG for the general function prediction only, followed by signal transduction mechanisms, post-translational modification, protein turnover, and chaperones (Figure S1a). Based on species distribution analysis according to the NR database, the majority of annotated genes were found to match *P. sitchensis*, followed by *Quercus suber*, *Amborella trichopoda*, and *Carpinus fangiana* (Figure S1b).

Next-generation sequencing (NGS) was performed using the DNBSEQ sequencing platform and uploaded to NBCI (PRJNA1202384). Six samples from the stem of *P. massoniana* were divided into two groups: CK (3 h after treatment with Tween-20) and T (3 h after treatment with MeJA). Each sample produced an average of 8.29 Gb of clean reads, with an average gene set alignment rate of 83.73%, resulting in the detection of 383,514 genes. The lowest Q30 values obtained for each group were 89.43% (CK) and 89.77% (T), and the error rate was below 0.69%. On average, 55.27 million clean reads were obtained per sample. The mapping ratios to the reference gene ranged from 82.15% to 87.27%, indicating a strong match and reliable data quality (Table S2).

### 2.2. DEG Identification

The criterion |log_2_(FoldChange)| > 2 was used as the standard for pairwise differential expression analysis of CK and T sample groups. Principal component analysis (PCA) showed significant differences between the CK and T sample groups (Figure S2a). The analysis identified 12,022 DEGs (6154 upregulated, 5858 downregulated) in the CK vs. T comparison (Figure S2b).

**Figure 2:**
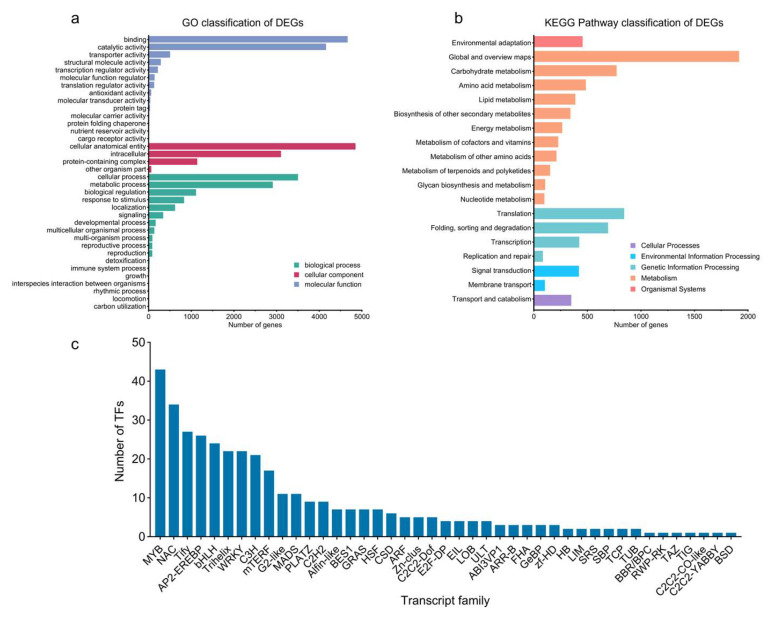
DEG identification of MeJA treatment transcriptome of *P. massoniana*. (**a**) GO classification of DEGs. (**b**) KEGG pathway classification of DEGs. (**c**) Transcript family analysis of DEGs.

Based on the GO enrichment results, the DEGs obtained in the CK vs. T comparison can be divided into three categories: biological process, cellular component, and molecular function (Figure 2a). The most highly enriched items are the cellular anatomical entity (4852) in the biological process and binding (4667) in the molecular function, which are related to the antioxidant and enzymatic activities in plants, respectively. The cellular component indicates the most DEGs of the three function classes, and the most enriched items are the cellular anatomical entity, intracellular, and protein-containing complex, while the other organism component indicates the fewest annotated DEGs. In the biological process class, cellular processes and metabolic processes show the most DEGs. In the molecular function class, binding and catalytic activity are the most annotated DEGs. After MeJA treatment, 4829 DEGs were mapped to 133 Level 3 KEGG pathways, 19 of which were significantly enriched compared to the control group (Figure 2b). Most of these pathways fall under ‘Metabolism’ at Level 1, with a focus on ‘Metabolism of terpenoids and polyketides’ at Level 2. Other enriched pathways include biosynthesis of secondary metabolites, carbohydrate metabolism, environmental adaptation, folding, sorting, degradation, energy metabolism, metabolism of other amino acids, lipid metabolism, signal transduction, transcription, and translation, all of which are crucial for basic plant growth, development, and secondary defense metabolism (Table S3).

### 2.3. Transcription Factor Analysis of DEGs

A total of 376 transcription factor types were identified, with the MYB family representing the largest group (43 transcription factors), followed by the NAC (34), Tify (28), AP2-EREBP (26), bHLH (24), and WRKY (22) families. Smaller families, including Trihelix, C3H, mTERF, and others, were also identified, while the TIG, TAZ, RWP-RK, C2C2-YABBY, C2C2-CO-like, BSD, and BBR/BPC families contained only one transcription factor each (Figure 2c).

### 2.4. Identification of bHLH Transcription Factors of DEGs in P. massoniana

**Figure 3:**
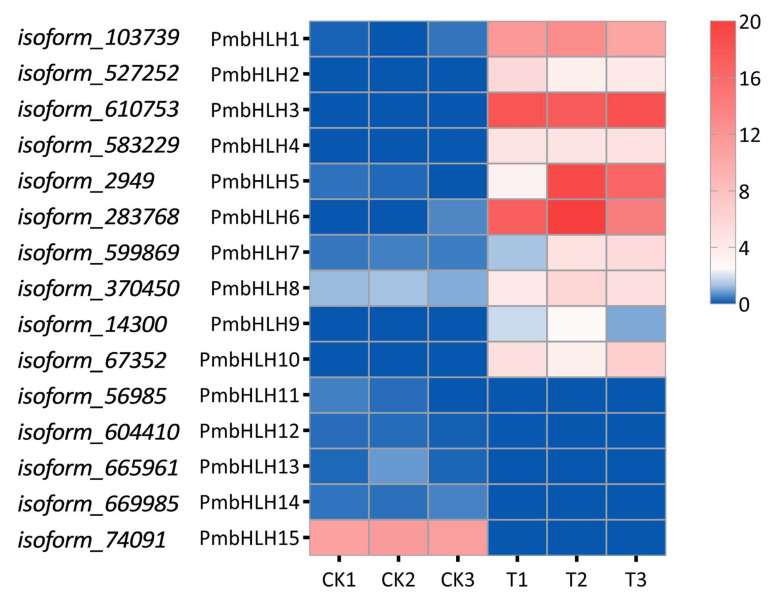
The heat map of 15 different expressed bHLH transcription factors in *P. massoniana*.

A total of 15 differential expression bHLH transcription factors were selected with |log_2_(FoldChange)| > 2 and verified using the CD-search program in *P. massoniana*. These genes were named from PmbHLH1 to PmbHLH15. The number of amino acids in the predicted protein products varied from 237 to 801 (Table S4). Among the 15 PmbHLH proteins, PmbHLH1, with 237 amino acids, was the smallest, while the largest protein was PmbHLH15 (801 amino acids). The range of protein molecular weights was 26.38–87.15 kDa, and the isoelectric point values ranged from 5.45 (PmbHLH5) to 9.14 (PmbHLH15). According to the results of the Wolf PSORT prediction of the subcellular localization of PmbHLH1-PmbHLH15 proteins, almost all the proteins have a predicted nuclear localization. Heat maps were generated using the FPKM values of the 15 differential bHLH transcription factors. The results showed that the expression levels of PmbHLH1 to PmbHLH10 increased after treatment, with PmbHLH3, PmbHLH5, and PmbHLH6 upregulated approximately 20-fold. In contrast, the expression of PmbHLH11 to PmbHLH15 decreased, with PmbHLH15 showing the largest downregulation, approximately 10-fold (Figure 3).

**Figure 4:**
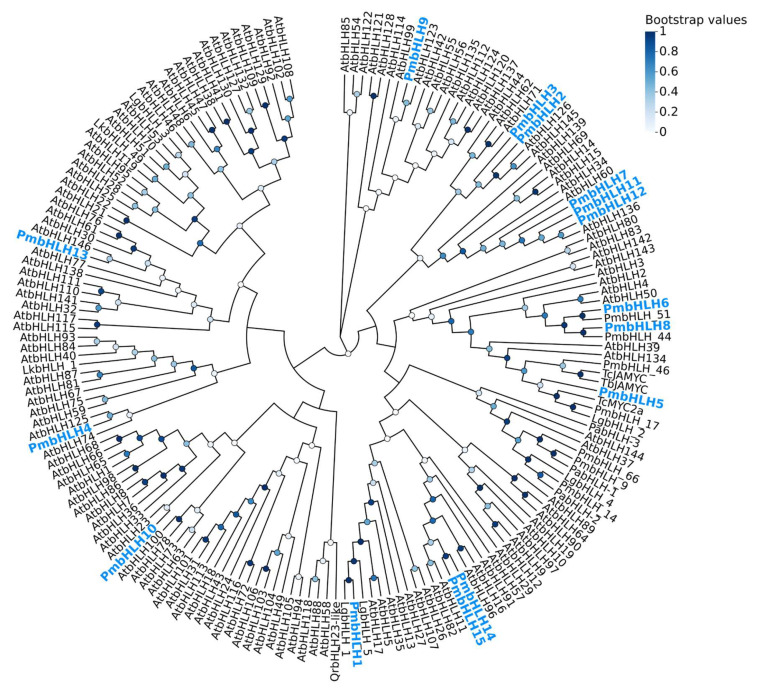
A phylogenetic tree of 15 *P. massoniana* bHLH transcription factor proteins (in blue font), together with 146 A. thaliana bHLH transcription factor proteins and 22 conifer bHLH proteins. The accession numbers of all AtbHLH proteins and other bHLH proteins are given in Supplementary Table S5. Missing data and positions with gaps were eliminated. The circles on the branches correspond to the bootstrap support (1000 replications).

The neighbor-joining (NJ) method was used to construct a phylogenetic tree of 15 PmbHLH protein sequences from *P. massoniana*, 146 AtbHLH protein sequences from Arabidopsis thaliana, and 22 conifer bHLH proteins (Figure 4). The results showed that PmbHLH7, PmbHLH11, and PmbHLH12 are closely related to AtbHLH136. PmbHLH6 and PmbHLH 51 form a branch together, while PmbHLH8 and PmbHLH 44 also cluster into a distinct branch, both forming two highly supported sub-branches within the main clade. PmbHLH5 shares a close relationship with TcMYC2A from Taxus chinensis, LgbHLH2 from Larix gmelinii var. olgensis, and PmbHLH 17 from *P. massoniana*, suggesting that PmbHLH5 may belong to the MYC subfamily of the bHLH transcription factor family. PmbHLH14 and PmbHLH15 are grouped in a strongly supported sub-branch, closely associated with AtbHLH11 and AtbHLH82. PmbHLH1 and LgbHLH1 are clustered together and show strong homology with LgbHLH5. In contrast, PmbHLH4, PmbHLH9, PmbHLH10, and PmbHLH13 are distantly related to other PmbHLHs, each forming a separate branch with AtbHLH125, AtbHLH123, AtbHLH23, and AtbHLH146, respectively.

### 2.5. Protein Motif Analysis

**Figure 5:**
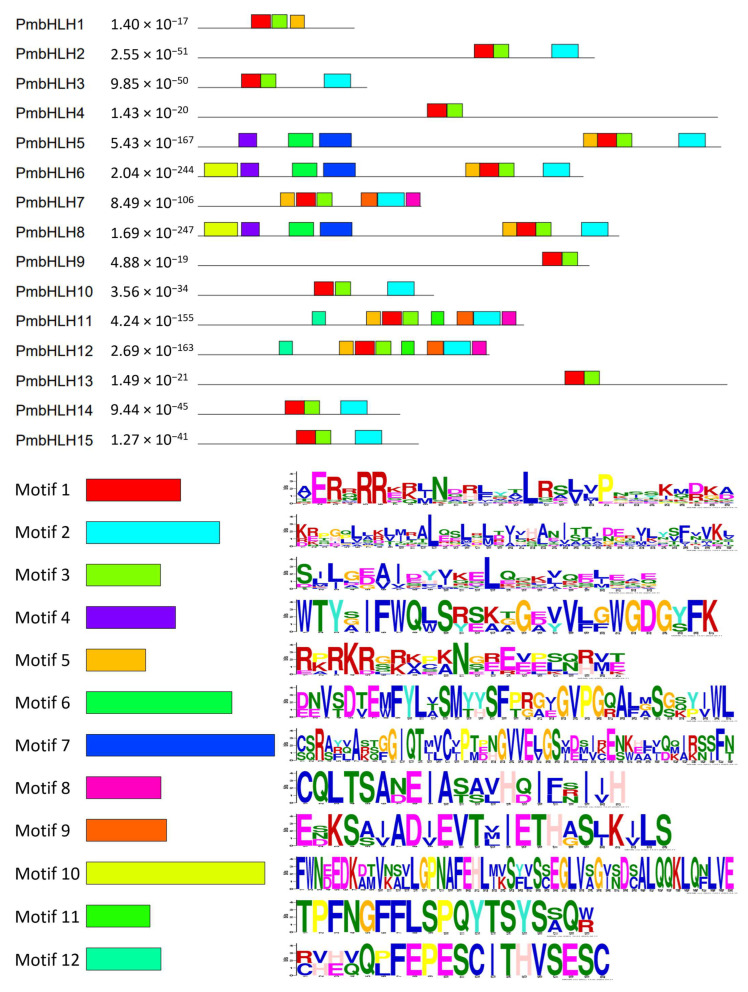
Motif analysis of bHLH transcription factors in *P. massoniana*.

The motif types and permutations of PmbHLHs in *P. massoniana* (Figure 5) were analyzed using the online Multiple EM for Motif Elicitation (MEME) software. The number and species of the 12 predicted motifs ranged from 19 to 50 aa in length. The bHLH domain consists of two functional regions: the N-terminal basic amino acid region and the C-terminal HLH region. These regions correspond to motif 1 and motif 3, respectively. All PmbHLH1–PmbHLH15 genes contain both motif 1 and motif 3. In addition to motifs 1 and 3, 11 PmbHLHs have motif 2, except for PmbHLH1/4/9/13. It is typically located at the C-terminal relative to motifs 1 and 2, although not necessarily in a continuous sequence. Additionally, PmbHLH2/3/10/14/15 share a similar predictive motif, suggesting they belong to the same class of transcription factors within the bHLH family. Similarly, PmbHLH6/8, PmbHLH11/12, as well as PmbHLH4/9/13, showed pairwise similarity in their motif structures. Motif analysis of the conifer bHLH sequence combination downloaded by NCBI was also performed (Figure S3). The results showed that PmbHLH5/6/8 had the same motif as TcJAMYC and LgbHLH-2. Similarly, QrbHLH23-like, LkbHLH-1, LkBHLH-49, and pm4/9/10/13/14/15 also have the same motif composition.

### 2.6. Yeast One-Hybrid (Y1H) Assay of the Interaction of PmbHLHs with Pm TPS (−)-α-Pinene Promoter

**Figure 6:**
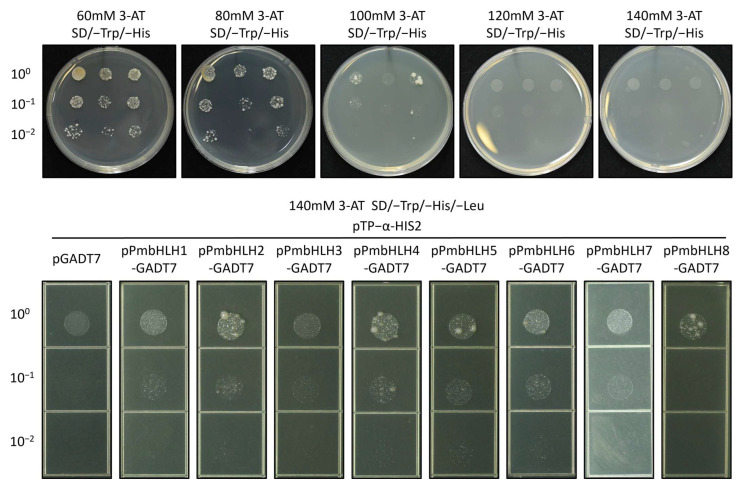
Identification of the minimum inhibitory concentration of 3-AT and yeast one-hybrid assay of PmbHLH1/2/3/4/5/6/7/8 (pPmbHLH1-8) with Pm TPS (−)-α-pinene promoter (pTP−α-HIS2) on SD/–Trp/–His/–Leu/140mM 3-AT plates.

The BD vector was constructed using the TPS (−)-α-pinene promoter fragment from *P. massoniana*. The successfully constructed vector, named pTP−α-HIS2, was used for point-to-point verification in a Y1H assay. To omit the influence of target sequence recognition by endogenous yeast transcription factors, the minimum inhibitory concentration of 3-AT (basal expression level of reporter gene) was measured. As shown in Figure 6, the minimum concentrations of 3-AT needed to suppress basal expression of the TP−α bait strains were 140 mM.

The AD vector was constructed using the ORF region of PmbHLH genes, and the successfully constructed vector was named pPmbHLH1/2/3/4/5/6/7/8-GADT7. Y1H assay was conducted on SD/–Trp/–His/–Leu/140mM 3-AT plate. The results showed moderate interactions between PmbHLH2, PmbHLH4, PmbHLH5, PmbHLH6, and PmbHLH8 with the promoter fragment of the *P. massoniana* TPS (−)-α-pinene promoter. PmbHLH8 exhibited a strong interaction at a high concentration (100) but no interaction at lower concentrations (10^−1^, 10^−2^). No significant interaction was detected with PmbHLH1, PmbHLH3, or PmbHLH7 (Figure 6). The picture has been partially cropped, and the complete image can be found in the Supplementary Figure S4.

### 2.7. Subcellular Localization Analysis

**Figure 7:**
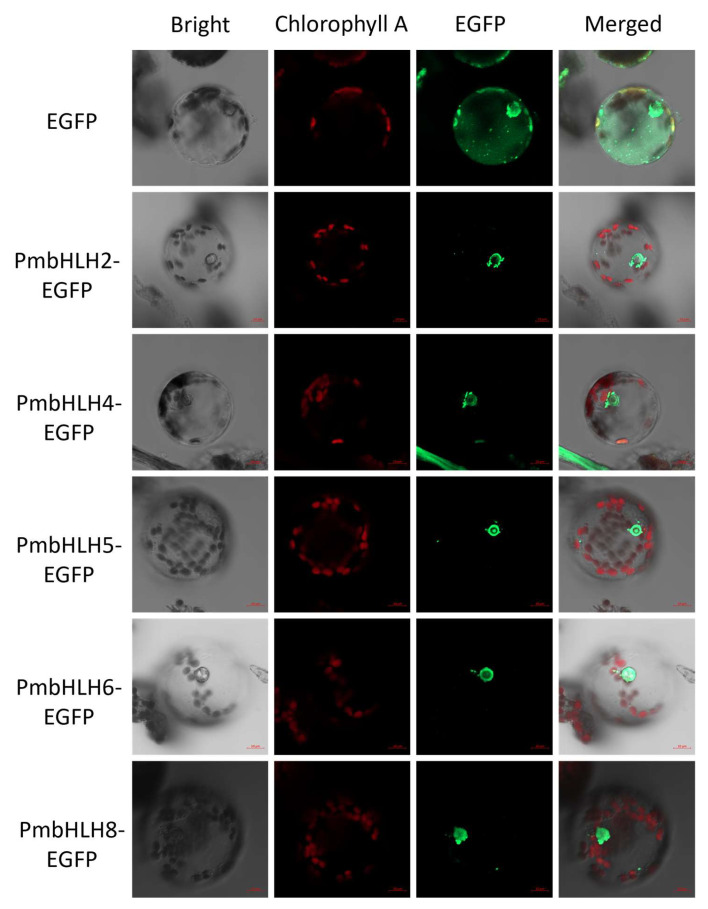
Subcellular localization assays of PmbHLH2/4/5/6/8 proteins in *P. massoniana* protoplasts. The scale bar is 10 μm.

According to the results of the Wolf PSORT prediction of the subcellular localization of PmbHLH1–PmbHLH15 proteins, almost all the proteins have a predicted nuclear localization. To confirm the subcellular localization of PmbHLH transcription factors interacting with the *P. massoniana* TPS-(−)-α-pinene promoter fragment, primers were designed based on the coding sequences of PmbHLH2/4/5/6/8 to construct subcellular localization vectors. The PmbHLH1/3/7 transcription factors were excluded from the subcellular localization analysis due to the absence of interaction in the Y1H experiment. The constructed vector, named pBI121-EGFP-PmbHLH2/4/5/6/8, was introduced into *P. massoniana* protoplast. Microscopic analysis of protoplasts using a hemocytometer revealed a viability of 98%, with a fragmentation rate of less than 10% (Figure S5). Confocal microscopy revealed that the EGFP fluorescence was localized exclusively in the nucleus, whereas fluorescence of the control EGFP was distributed throughout the cells, confirming that these five bHLH transcription factors are nuclear-localized proteins (Figure 7). The EGPF fluorescence was partially enhanced for better visualization.

### 2.8. Gene Expression Analysis Under MeJA and M. alternatus Adult Feeding Treatment

**Figure 8:**
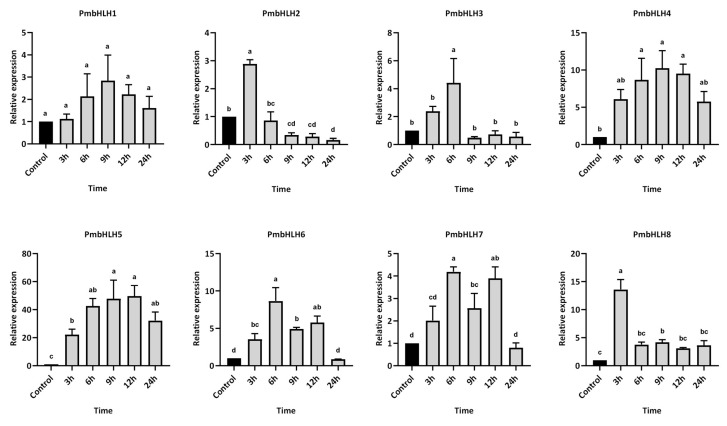
The relative expression of bHLH DEGs in *P. massoniana* after MeJA treatment, verified by qPCR. Bars with different letters indicate significant differences (*p* < 0.05). The relative expression of the control group was defined as 1.

To investigate the temporal expression of PmbHLH genes in *P. massoniana* following MeJA treatment, eight differentially expressed PmbHLH genes with high induction levels were selected for expression analysis within the first 24 h post-treatment (Figure 8). Overall, MeJA treatment significantly induced the expression of these bHLH genes in the *P. massoniana* branches, with each gene reaching its peak expression at a distinct time point. Among them, PmbHLH1 exhibited a gradual increase in expression, though changes were not statistically significant compared to the control group throughout the 24 h period. PmbHLH2 and PmbHLH8 were rapidly upregulated within 3 h, reaching approximately 3-fold and 13-fold the control levels, respectively. Both subsequently declined, falling below control levels by the end of 6 h post-treatment. PmbHLH3, PmbHLH6, and PmbHLH7 peaked at 6 h, with expression levels approximately 4-fold, 8-fold, and 4-fold higher than controls, respectively. While PmbHLH3 expression dropped below control levels after 9 h, PmbHLH6 and PmbHLH7 remained elevated and returned to baseline only after 24 h. PmbHLH4 and PmbHLH5 showed similar temporal patterns, with expression increasing at 3 h and sustaining elevated levels through 24 h. Their peaks occurred at 9 h, with PmbHLH4 and PmbHLH5 reaching about 10-fold and 50-fold higher expression than the control, respectively.

**Figure 9:**
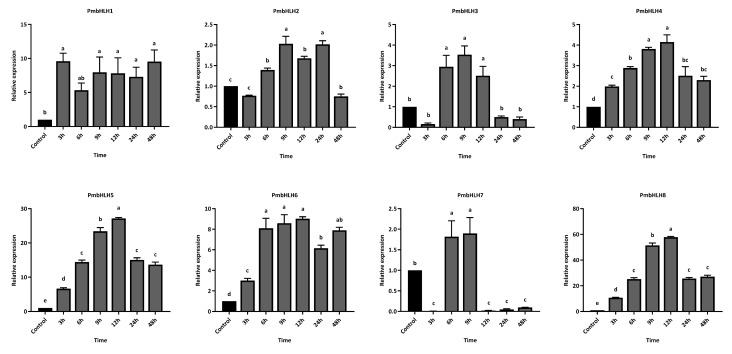
The relative expression of bHLH DEGs in *P. massoniana* under *M. alternatus* feeding, verified by qPCR. Bars with different letters indicate significant differences (*p* < 0.05). The relative expression of the control group was defined as 1.

Furthermore, we measured the temporal expression levels of these eight highly upregulated PmbHLH DEGs at different time points after *M. alternatus* adult feeding. Within 48 h of feeding treatment, the PmbHLH1 to PmbHLH8 genes were significantly upregulated in the stems of *P. massoniana*, with each gene reaching peak expression at distinct time points (Figure 9). The expression of PmbHLH1 peaked at 3 h post-treatment, reaching nearly 10 times that of the control group, and remained elevated throughout the 48-hour period. The expression levels of PmbHLH4, PmbHLH5, and PmbHLH8 increased consistently after treatment, peaking at 12 h before declining within the 48-hour timeframe. Notably, the expression of PmbHLH4 increased 4-fold compared to the control, whereas PmbHLH5 and PmbHLH8 demonstrated significantly higher increases, rising 27-fold and 57-fold, respectively, within the same 12-hour period. In contrast, PmbHLH2, PmbHLH3, PmbHLH6, and PmbHLH7 reached peak expression between 6 and 12 h after treatment, respectively. Among these, the expression levels of PmbHLH2, PmbHLH3, and PmbHLH7 increased by approximately twofold, while PmbHLH6 showed a more substantial ninefold increase within 6 h. PmbHLH3 expression decreased to control levels at both 3 h and 24 h after, while PmbHLH7 expression dropped below control levels at 3 h and again at 12 h after.

## 3. Discussion

Previously, we demonstrated that exogenous MeJA treatment and *M. alternatus* adult feeding can induce resin-based defense in *P. massoniana*, including the formation of secondary resin ducts, increased resin terpenoid accumulation, and changes in the expression of terpene synthase genes involved in metabolic pathways [4,75,79]. In this study, we further identified 15 *bHLH* differentially expressed genes (PmbHLH1 to PmbHLH15) of *P. massoniana* through the MeJA treatment (simulated feeding) transcriptome. In addition, PmbHLH1 to PmbHLH8, which were highly expressed post MeJA treatment, also showed temporal expression changes due to *M. alternatus* feeding treatment, among which five of the *PmbHLH* genes were identified as Pm TPS (−)-*α*-pinene promoter interacting transcription factors through the Y1H assay. We suggest that these genes are involved in the regulation of *α*-pinene of *P. massoniana* under biotic stress.

The bHLH gene family is widely distributed in both plants and animals, constituting one of the largest transcription factor families in plants [26,87,88]. A recent study reported 88 bHLH-encoding proteins in the *P. massoniana* transcriptome [89], which is fewer than typically found in angiosperms [90,91]. In the present study, 744 *bHLH* gene annotation isoforms were identified from the transcriptome data, among which 78 were differentially expressed, and 15 highly differentially expressed *bHLH* genes were selected. Notably, we found many duplicate isoforms coding for the same *bHLH* gene during the candidate genes screening, which, despite the absence of genome-wide replication, may be related to the reproducibility of the Pinaceae genome [92]. qPCR analysis revealed that the expression levels of most identified bHLH genes increased rapidly following feeding or simulated feeding by *M. alternatus*, with peak expression observed within 1–2 days after treatment. This temporal pattern closely mirrored that of key synthase genes involved in terpene-based defense, suggesting that *P. massoniana* initiates a timely and coordinated regulation of secondary metabolism in response to herbivory, characterized by rapid yet well-organized gene expression dynamics [4,93]. In Arabidopsis, plants overexpressing the bHLH gene exhibited a slight increase in bHLH expression during the first 6 h under long-day conditions, followed by a significant rise, peaking at 9–24 h, with expression levels approximately 20 times higher than those of wild-type plants [94]. In *Dendrobium huoshanense*, bHLH genes showed the highest expression level of *DhbHLH* at 4 and 16 h after MeJA treatment and may be involved in the synthesis of alkaloids induced by JA [95]. Additionally, a study from *Betula platyphylla* demonstrated that the expression of *BpbHLH9* increased 5.2-fold following 12 h after MeJA treatment but declined to levels below the control group after 24 h, and it was involved in the biosynthesis of triterpenoids [96]. These studies suggest that bHLH genes in angiosperms respond more quickly to the induced defense compared to conifers. Interestingly, peak bHLH expression in *Passiflora edulis* and *Areca catechu* in response to abiotic stress was also observed 1–2 days post-treatment [97,98], providing further insights into the temporal dynamics of bHLH expression under both biotic and abiotic stress.

The core DNA-binding domain of the bHLH protein includes an alkaline region that recognizes and binds the core hexanucleotide sequence [99]. The amino acid sequences in this region are key to distinguishing the bHLH family [100,101]. In this study, it was found that the conserved domains of a part of PmbHLH genes were nearly identical. We classified the PmbHLHs into four groups based on their domain distribution patterns: The first group, consisting of PmbHLH4, PmbHLH9, and PmbHLH13, contains only the typical DNA-binding domain (motif 1 and motif 3). The second group, including PmbHLH2, PmbHLH3, PmbHLH10, PmbHLH14, and PmbHLH15, contains both the DNA-binding domain and the C-terminal motif 2. The third group, comprising PmbHLH5, PmbHLH6, and PmbHLH8, contains motif 4, motif 11, motif 7, motif 5, and all motifs present in the second group. The fourth group, characterized by PmbHLH7, PmbHLH11, and PmbHLH12, is distinguished by the presence of additional motif 9 and motif 8 flanking motif 2. There were significant differences in conserved domains between different PmbHLH groups. This pattern was further supported by another transcriptome study of PmbHLH [89] and is notably similar to the bHLH transcription factors found in apple (*Malus × domestica* Borkh.) [102]. Additionally, ZmbHLH in *Zea mays* within the same group displayed similar genetic structures and conserved protein motifs, suggesting that members of the same group in the bHLH family are closely related evolutionarily [103]. This may explain why the functions of bHLH proteins are often group-specific. Notably, motif analysis of conifer bHLH proteins revealed that the motif composition of PmbHLH was similar to TcbHLH (*Taxus chinensis*) and QrbHLH (*Quercus robur*) but differed from that of *P. abies*, another Pinaceae species. This contrasts with the evolutionary pattern observed in other proteins, such as A. thaliana subfamily Ia proteins [104]. Pires et al. previously proposed the monophyletic evolution of bHLH proteins [68], and our findings provide further support for this hypothesis. Outside of the bHLH domain, the amino acid sequences of PmbHLH proteins are poorly conserved, and the length of the encoded proteins varies, for example, PmbHLH1 and PmbHLH5. The variability in sequences outside the functional domain of bHLH transcription factors contributes to a complex evolutionary model, which may lead to low support values for branches in the phylogenetic tree. This variability is also observed in other plants, where additional domains are present to regulate their activity and/or DNA-binding specificity [67,105,106].

Subcellular localization is a reliable method for determining whether a gene functions as a transcription factor [107]. In the current study, transient expression of GFP fusion proteins in tobacco leaf or onion epidermal cells was used as a convenient and effective approach [108,109]. However, due to species-specific limitations, subcellular localization in non-native species may affect GFP fusion protein expression and transfer [110]. Therefore, subcellular localization experiments were conducted in *P. massoniana* protoplasts in this study. Notably, PmbHLH5 appears to exhibit distinct nucleolar localization. The possibility of detecting nucleolar protein localization by GFP fusion protein has been demonstrated [111]; however, whether this is related to the specific transfer process of fusion protein remains to be verified [112].

In this study, 15 differentially expressed bHLH proteins were identified from the MeJA-treated transcriptome of *P. massoniana*, suggesting that the bHLH transcription factors are associated with JA-mediated defense under simulated feeding conditions. However, whether bHLHs are involved in the regulation of other pathways in *P. massoniana* induced by herbivore feeding remains to be investigated. The interaction of five highly expressed bHLH genes with the Pm TPS (−)-*α*-pinene promoter has been confirmed through yeast one-hybrid assays; however, their functions remain unverified. Further experiments, such as enzyme activity analysis and transgenic experiments, are needed to determine whether these genes positively regulate Pm TPS (−)-*α*-pinene gene transcription or enhance the JA-induced terpenoid defense response in *P. massoniana*.

## 4. Materials and Methods

### 4.1. Plant and Insect Materials

*P. massoniana* seeds were purchased from the Paiyangshan Forest Centre in Chongzuo, Guangxi Province (22°14′ N, 107°04′ E), approved by the local authorities, and then stored in the College of Forestry and Grassland, Nanjing Forestry University. The seeds were soaked in warm water at 40 °C for 8 h and then spread in an autoclaved nutrient medium (humus/turf soil/perlite, 3:1:1 by volume). The seedlings were cultivated in the greenhouse (26 ± 0.5 °C, relative humidity = 70 ± 5%, 16:8 h light/dark photoperiod) of Nanjing Forestry University.

We obtained permission from local authorities to collect the two-year-old *P. massoniana* seedling from Pingxiang, Jiangxi Province (26°76′ N, 114°28′ E) in China, which were then planted individually in a greenhouse (26 ± 0.5 °C, relative humidity = 70 ± 5%, 16:8 h light/dark photoperiod) of Nanjing Forestry University with nutrient medium (humus/turf soil/perlite, 3:1:1 by volume) [4]. The seedlings were watered weekly. The third-instar larvae of *M. alternatus* were collected from *P. massoniana* host trees in Quanjiao County, Anhui Province, China (41°31′ N, 117°74′ E). Subsequently, they were reared individually on an artificial feed at a constant temperature of 26 ± 0.5 °C, relative humidity = 60 ± 5%, and a 16:8 h light/dark photoperiod, as described by Chen et al. [4]. Newly emerged *M. alternatus* adults were collected and individually kept in 250 mL glass flasks, provisioned with fresh *P. massoniana* twigs as food in climate-controlled rooms (26 ± 0.5 °C, relative humidity = 60 ± 5%, 16:8 h light/dark photoperiod). Adult beetles (a mixture of males and females) visually estimated to be of similar physiological conditions and age close to 15 days ±3 days were chosen and starved at room temperature for 24 h before use. Finally, the plant materials were authenticated by Prof. Kongshu Ji from Nanjing Forestry University, China. The voucher specimen (NF1001839) was deposited at the Dendrological Herbarium, Nanjing Forestry University (Institution code from Chinese Virtual Herbarium: NF).

### 4.2. MeJA Treatment and Feeding Treatment

Each seedling was sprayed with MeJA (Sigma-Aldrich, St. Louis, MO, USA; 95% purity) dissolved in 0.1% (*v*/*v*) Tween-20 solution for a final concentration of 10 mM (150 mL) over a period of 30 min as the treatment group. Each seedling was sprayed with 0.1% (*v*/*v*) Tween-20 solution (150 mL) as the control group. The time point at the end of spraying was denoted as 0 h. At 0 h, 3 h, 6 h, 9 h, 12 h, 24 h, and 48 h after treatment, the stem parts above the ground and below the upper internode of the seedlings (discarded below 3 cm above the ground) were cut off and collected, respectively. Both groups of seedlings were sprayed at the same time and then separated to prevent crosstalk between the groups.

The feeding treatment was administered according to Wen et al. [75]. Healthy *P. massoniana* seedlings under similar growth conditions were selected and divided into treatment and control groups. The starved adults of *M. alternatus* were caged individually on a seedling stem using a wire mesh (25 cm × 25 cm, hole size = 4 mm^2^) surrounding the stem of *P. massoniana* seedlings, and the feeding time was recorded. Each seedling was inoculated with one *M. alternatus* adult. Each group had three biological replicates. A total of six groups were set up, namely, after feeding for 3 h (hours), 6 h, 9 h, 12 h, 24 h, and 48 h, the wire mesh and the insects were removed (N = 3). The control group did not receive any treatment. Subsequently, the seedlings of the treatment and control groups were sampled. The stems of *P. massoniana*, 5 cm above and below the feeding center of *M. alternatus*, were cut into small sections (1–2 cm long). The sampled stems were transferred to ribonuclease-free centrifuge tubes, quickly frozen in liquid nitrogen, and stored at −80 °C until use. 

### 4.3. RNA Isolation and Assessment

Total RNA was extracted from the stem tissue of *P. massoniana* using a Miniprep RNA Purification Kit (TIANGEN, Beijing, China). Using agarose gel electrophoresis and spectrophotometry (Nano Drop ND-1000, Thermo Fisher Scientific, Waltham, MA, USA), total RNA was measured. Total RNA was reverse-transcribed to synthesize complementary deoxyribonucleic acid (cDNA) using a 5× All-In-One RT Master Mix (Accurate Biology, Changsha, China).

### 4.4. NGS Library Construction and Sequencing

The RNA samples of *P. massoniana* treated with MeJA (named “T”) and Tween-20 (named “CK”) for 3 h were used for NGS library construction. Total RNA was processed using an mRNA enrichment method. For mRNA enrichment, magnetic beads coated with Oligo (dT) were used to selectively bind and isolate mRNA molecules with poly (A) tails. In the rRNA removal method, rRNA was hybridized with DNA probes, and the resulting RNA-DNA hybrids were selectively digested using RNase H. Residual DNA probes were then eliminated with DNase I, leaving the desired RNA for further processing. The purified RNA was fragmented using an interrupting buffer and reverse-transcribed into cDNA using random N6 primers. Double-stranded cDNA was synthesized to produce double-stranded DNA. These DNA fragments were end repaired, phosphorylated at the 5′ ends, and had “A” overhangs added at their 3′ ends. A sequencing adapter with a “T” overhang was ligated to these fragments. The ligated products were amplified via PCR using specific primers. The PCR products were denatured to yield single-stranded DNA, which was circularized using a bridge primer to form a single-stranded circular DNA library. The sequencing library was then analyzed using the DNBSEQ platform.

### 4.5. Full-Length Transcriptome Library (PacBio) Preparation and Sequencing

Total RNA was extracted from the stem tissues of eight samples, including 0 h, 3 h, 6 h, 9 h, 12 h, 24 h, and 48 h of MeJA treatment and control treatment. Almost equal amounts of high-quality RNA were mixed to generate an informative reference transcript database. DNA library construction and sequencing with the PacBio Sequel II platform were performed by Huada Gene and Biological Company, Guangzhou, China. The PacBio full-length transcriptome library preparation involves constructing SMRTbell libraries from total RNA in two size ranges (0–5 K and 5–10 K) for sequencing on the PacBio platform. The process includes sample quality control (QC) to assess RNA concentration, integrity, and purification, followed by reverse transcription to generate full-length cDNA. For the 4.5–10 K library, DNA fragments are size selected using BluePippin and PCR amplified. Both library types undergo end repair, SMRTbell adapter ligation, and enzyme digestion to repair damaged or linear DNA without adapters. Libraries are validated through QC before being loaded onto chips for sequencing.

### 4.6. Transcriptome Data Processing

The identification and annotation of full-length transcripts were conducted using the SMRT-Analysis software package (smrtlink8.0, PacBio, Menlo Park, CA, USA). Iso-Seq data analysis included recognizing reads of insert (smrtlink ccs), identifying full-length transcripts (blastn2.2.28: -outfmt 7 -word_size 5), clustering (isoseq3 cluster), and correcting (isoseq3 polish) to produce consistent full-length sequences. Transcripts from all samples were merged and re-clustered to refine the dataset. High-quality transcripts were assessed using BUSCO [113] (v3.0.1) and functionally annotated across seven databases, including Pfam [114], NT, NR, KOG [115], KEGG [116], Swiss-Prot [117], and GO [118], with tools such as hmmscan [119], Blastn [120] (accessed on 13 January 2023), Blastx [120], Diamond [121], and Blast2GO [122]. Transcript quantification and differential expression analysis were based on non-redundant transcript clustering across samples. Transcript expression levels were calculated as a proportion of cluster reads, and DESeq2 [123] was used to identify significant differences between groups, applying thresholds of Q ≤ 0.05 and |Log_2_FoldChange| ≥ 2. Differentially expressed genes were visualized with hierarchical clustering using the pheatmap package in the R programming language (Version 4.4.3). Enrichment analysis for KEGG pathways and GO terms was performed using R’s phyper function and GO-TermFinder [124], with corrected *p*-values ≤ 0.01 indicating significance. Coding sequences (CDS) were predicted using Transdecoder [125], which identified open reading frames and aligned them to the Swissprot and Pfam databases. This process culminated in precise predictions of transcript coding regions. Simple sequence repeats (SSRs) were detected with MISA [126], and primers were designed with Primer3 [127]. Primers underwent a rigorous screening process to ensure specificity and alignment to target SSR regions, validated further by SSRFinder. To predict the coding potential of novel transcripts, CPC [128], txCdsPredict, CNCI [129], and the Pfam database were employed. Transcripts were classified as mRNA or lncRNA based on consistent results from at least three of the four methods. Thresholds for classification included CPC and CNCI scores of >0 for mRNA, txCdsPredict values > 500 for mRNA, and Pfam database mapping for additional verification. This comprehensive approach ensured accurate transcript classification.

To quantify the number of transcription factors in differentially expressed genes, Getorf was used to detect the open reading frames (ORFs) for each isoform, followed by Hmmsearch to match these ORFs with known transcription factor protein domains. The isoforms were then categorized based on the characteristics of their respective transcription factor families.

### 4.7. Bioinformatics Analysis

The online platform ExPASY (https://web.expasy.org/compute_pi/, accessed on 1 December 2024) was used to calculate the isoelectric point, molecular weight, and hydrophobic/hydrophilic prediction. The subcellular localization of proteins was predicted using PSORT (https://www.genscript.com/psort.html, accessed on 1 December 2024). bHLH sequences of *A. thaliana* were downloaded from the TAIR database (https://www.arabidopsis.org/, accessed on 1 December 2024). The NJ (neighbor-joining) phylogenetic tree was constructed using MEGA (Version 7.0.26) with 1000 bootstrap replicates based on the bHLH proteins of *P. massoniana* and *A. thaliana* (Table S5). The phylogenetic tree was visualized using the online software chiplot (https://www.chiplot.online/, accessed on 1 December 2024) [130]. In addition, the online MEME software (Version 5.5.6) (https://meme-suite.org/meme/tools/meme, accessed on 26 December 2024) was used to predict and analyze the motifs of PmbHLHs. The motif width was set to 6–50, and the number of motifs was set to 20.

### 4.8. Y1H Assay

Eight upregulated *PmbHLH* genes with a threshold of log2 > 2 were selected to construct the vector. The Pm TPS (−)-*α*-pinene promoter (TP−*α*) sequence (1475 bp, accession number: LC860222) was amplified by PCR and inserted into the *EcoR*I/*Sac*I restriction sites of pHIS2 vector (carrying the HIS3 and TRP1 genes) to construct BD (DNA-binding domain) vector, and the ORFs of *PmbHLH* genes was amplified by PCR and inserted into the *Nde*I/*Xho*I restriction sites of pGADT7 vector (carrying LEU2 gene) to construct AD (activation domain) vector using a ClonExpress II One Step Cloning Kit (Vazyme, Nanjing, China). The recombinant product was transformed into *E. coli* DH5*α* competent cells and positive recombinant clones were screened using Luria–Bertani solid media containing ampicillin. The plasmids were then extracted and digested with EcoRI and SacI before PCR electrophoresis to confirm the insertion of the target fragment. The recombinant plasmids were identified by DNA sequencing. The recombined vector pHIS2-TP−*α* was transformed into the Y187 yeast strain using Yeastmaker™ Yeast Transformation System 2 (Clontech, Mountain View, CA, USA). The transformed cells were transferred onto SD/–Trp/–His solid media by adding 3-aminotriazole (3-AT) to determine the minimum inhibitory concentration.

The constructed AD vector and BD vector were transformed into Y187 yeast cells, respectively, and grew on the SD/–Trp/–His/–Leu/3-AT plates. Healthy single clones from SD/–Trp/–His/–Leu/3-AT plates were transferred into fresh SD/–Trp/–Leu medium and incubated at 30 °C for 3–5 days. The cultured yeast single clones were then transferred onto SD/–Trp/–His/–Leu/3-AT for the repeated test. The colonies that eventually grew on SD/–Trp/–His/–Leu/3-AT plates were selected for yeast colony PCR using a yeast colony PCR kit (Pronet Biotech, Nanjing, China). The product was identified by agarose gel electrophoresis.

### 4.9. Protoplast Transfection

Plasmid construction was performed using the ClonExpress II One Step Cloning Kit (Vazyme, Nanjing, China). The coding regions of the *PmbHLH* genes, excluding stop codons, were cloned into the plant binary expression vector pBI121-EGFP under the control of the cauliflower mosaic virus (CaMV) 35S promoter. Fifteen-day-old *P. massoniana* seedlings served as the starting material. Approximately 1 g of young needles were longitudinally sliced 2–3 times to enhance enzyme penetration. The tissue was incubated in 10 mL of enzyme solution (0.6 M mannitol, 20 mM MES (2-(N-morpholino) ethanesulfonic acid), 50 mM KCl, 10 mM CaCl_2_, 2%wt cellulase solution (Sigma-Aldrich), 0.125%wt pectinase solution (Sigma-Aldrich), and 0.1%wt BSA (Bovine serum albumin)) for 5 h in the dark at 28 °C. The reaction was halted by adding 10 mL of W5 solution (2 mM MES, 154 mM NaCl, 125 mM CaCl_2_, and 5 mM KCl), and undigested materials and debris were removed by filtering through a sterile 70 μm nylon mesh. The quality of protoplasts was measured by a hemocytometer. The resulting protoplasts were washed with W5 solution, then resuspended in MMG solution (4 mM MES, 5 mM KCl, 0.5 M mannitol, 15 mM MgCl_2_) at a concentration of 10^4^–10^6^ cells/mL. For transfection, 15 µg of plasmid was mixed with 100 µL of protoplast suspension and 100 µL of 30% PEG solution (30%wt PEG 4000, 0.2 M mannitol, 100 mM CaCl_2_) in a ribonuclease-free centrifuge tube. The mixture was incubated at room temperature in the dark for 30 min, followed by the addition of 1 mL of W5 solution to collect the protoplasts. Afterward, 100 µL of W1 solution (4 mM MES, 0.6 M mannitol, 20 mM KCl) was added, and the protoplasts were incubated at 28 °C in the dark for 16–24 h. Subcellular localization of fusion proteins was observed using confocal microscopy (Carl Zeiss, Oberkochen, Germany) and fluorescence microscopy with an excitation light source system (Lumen Dynamic Connections). All solutions used were sterilized using a 0.45 µm aqueous nylon filter. Each transfection was repeated at least three times to ensure reproducibility.

### 4.10. qRT-PCR

cDNA was synthesized from total RNA using the Hifair^®^ III 1st Strand cDNA Synthesis SuperMix (Yeasen, Shanghai, China) according to the recommended protocol. The qPCR primers are listed in Table S6. The cDNA templates diluted four times consecutively four times were used to construct a relative standard curve to determine the PCR efficiency, and all primers reached amplification efficiencies of 95–100%. qRT-PCR was performed in an Applied Biosystem 7900 System (Foster City, CA, USA) using Hieff^®^ qPCR SYBR Green Master Mix (Yeasen, Shanghai, China), according to the manufacturer’s protocol. The cycling conditions were as follows: 5 min at 95 °C; 40 cycles of 10 s at 95 °C and 40 s at 60 °C; and then, a melting curve analysis for continuous fluorescence monitoring while the sample was slowly heated from 60 to 95°C. Each reaction was run in triplicate, and the average threshold cycle (Ct) was calculated for each replicate. The gene expression levels were determined using the 2^−ΔΔCt^ method with ubiquitin-conjugating enzyme E2D (UBE2D) as the housekeeping gene. The relative expression of the control group was defined as 1.

Gene expression levels obtained via real-time quantitative PCR were first tested for normality using the Shapiro–Wilk test. Data not meeting the assumptions of normal distribution were transformed accordingly. One-way analysis of variance (ANOVA) was conducted to evaluate differences in gene expression at each time point relative to the control. Post hoc comparisons were performed using Tukey’s HSD test, with a significance threshold set at *p* < 0.05. Levene’s test was applied to assess the homogeneity of variances.

### 4.11. Statistical Analysis

All data were processed using SPSS 21.0 software, and the measurement data have been expressed as mean ± SE. Statistical differences between two groups were tested by Tukey HSD, and a *p* < 0.05 indicates a statistical significance.

## 5. Conclusions

In this study, 15 differentially expressed *PmbHLH* genes were identified in the MeJA-treated transcriptome of *P. massoniana*. Y1H assays demonstrated that five upregulated PmbHLHs—PmbHLH2, PmbHLH4, PmbHLH5, PmbHLH6, and PmbHLH8—interact with the Pm TPS (−)-*α*-pinene promoter. Subcellular localization experiments confirmed that these genes are expressed in the nucleus. Furthermore, qRT-PCR analysis revealed that eight highly upregulated PmbHLH genes were activated within two days after *M. alternatus* feeding, suggesting their involvement in *P. massoniana* resistance to *M. alternatus* and their temporal expression. The varying conserved domains of PmbHLH transcription factors not only validate the low conservation of extra domain sequences and the monophyletic evolution of the bHLH transcription factor family but also highlight potential target sites for future breeding programs aimed at developing resistant *P. massoniana* germplasm. These findings highlight the critical role of the *bHLH* gene family in *P. massoniana* defense mechanisms against herbivores and contribute to the understanding of terpene-induced defense regulation in host plants.

## Data Availability

All relevant data are contained within the article. The original contributions presented in the study are included in the article/Supplementary Materials; further inquiries can be directed to the corresponding authors. All the data have been deposited into the GenBank of the National Center for Biotechnology Information under accession numbers LC860222, PRJNA1205872, and PRJNA1202384.

## Supplementary Materials

The following supporting information can be downloaded at: https://www.mdpi.com/article/10.3390/plants14132038/s1, Figure S1. The annotation of transcripts in *P. massoniana* full-length transcriptome from different databases. Figure S2. DEG identification of MeJA treatment transcriptome of *P. massoniana*. Figure S3: Motif analysis of conifer bHLH sequence combination downloaded from NCBI and PmbHLHs. Figure S4: The protoplasts of Pinus massoniana were counted using a hemocytometer. Figure S5: Yeast one-hybrid of PmbHLH1/2/3/4/5/6/7/8 with Pm TPS (−)-α-pinene promoter on SD/-Trp/-His/-Leu/140mM 3-AT plates, respectively. Table S1: Unigenes annotation result statistics. Table S2: Summary of DNBSEQ sequencing quality. Table S3: KEGG pathway enrichment of DEGs in *P. massoniana*. Table S4: Features of bHLH transcription factor DEG-encoding sequences in *P. massoniana*. Table S5: Accession numbers of all AtbHLHs for the phylogenetic tree. Table S6: qPCR primers of PmbHLHs.

## Abbreviations

The following abbreviations are used in this manuscript:

Pm	*Pinus massoniana*
MeJA	Methyl jasmonate
bHLH	basic helix–loop–helix
NGS	Next-generation sequencing
DEG	Differentially expressed gene
TF	Transcription factor
Y1H	Yeast one-hybrid
TPS	Terpene synthase
3-AT	3-aminotriazole
EGFP	Enhanced green fluorescent protein
