# Supplementary material for: Full-Length Transcriptome Sequencing of Pinus massoniana Under Simulated Monochamus alternatus Feeding Highlights bHLH Transcription Factor Involved in Defense Response
Source: Plants (Basel). 2025 Jul 3;14(13):2038. doi: 10.3390/plants14132038 (PMC12251683; doi:10.3390/plants14132038)
Supplement: Supplementary file 1 [file plants-14-02038-s001.zip › Figure S1 S2 S3 S4 S5.pdf]

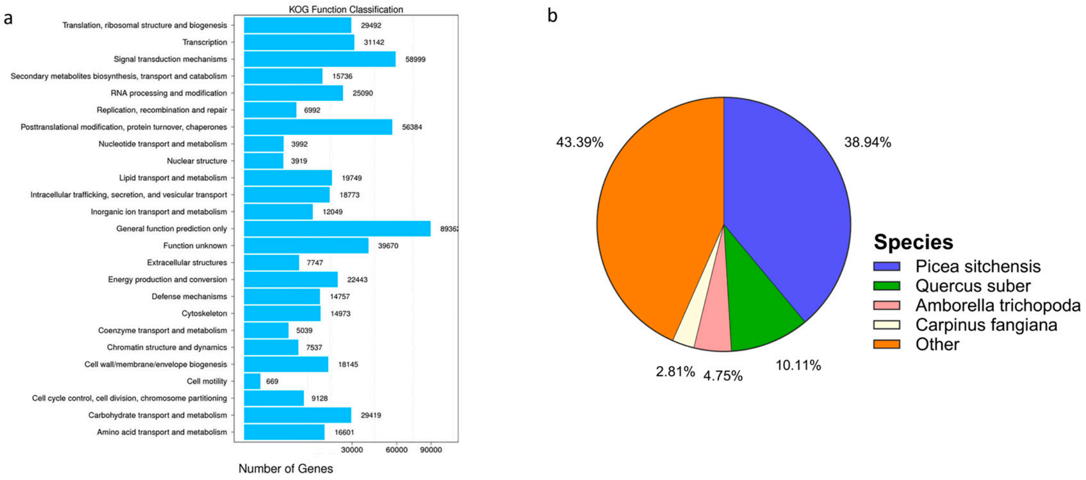

Figure S1. Annotation of transcripts in *P. massoniana* Full-length transcriptome from different databases. (a) KOG functional classification of *P. massoniana* transcriptome isoforms. (b) Species distribution analysis according the NR database of *P. massoniana* transcriptome isoforms.

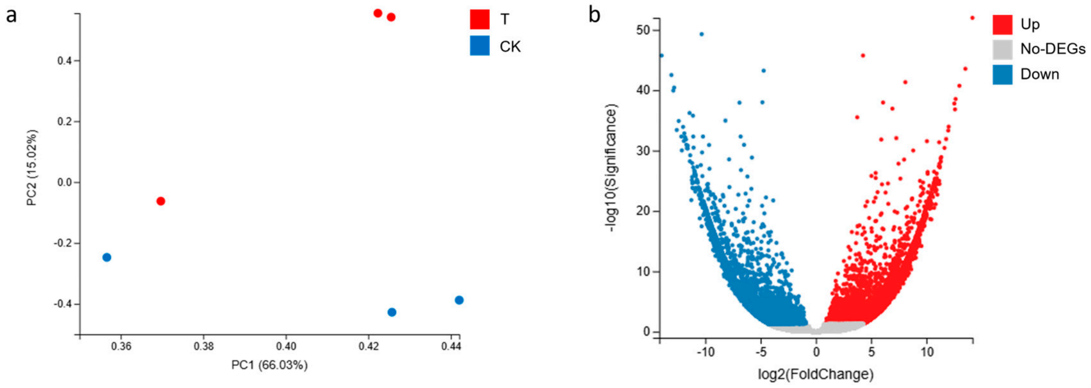

Figure S2. DEG identification of MeJA treatment transcriptome of *P. massoniana*. (a) Principal component analysis of CK and T sample groups. (b) DEGs volcano map.

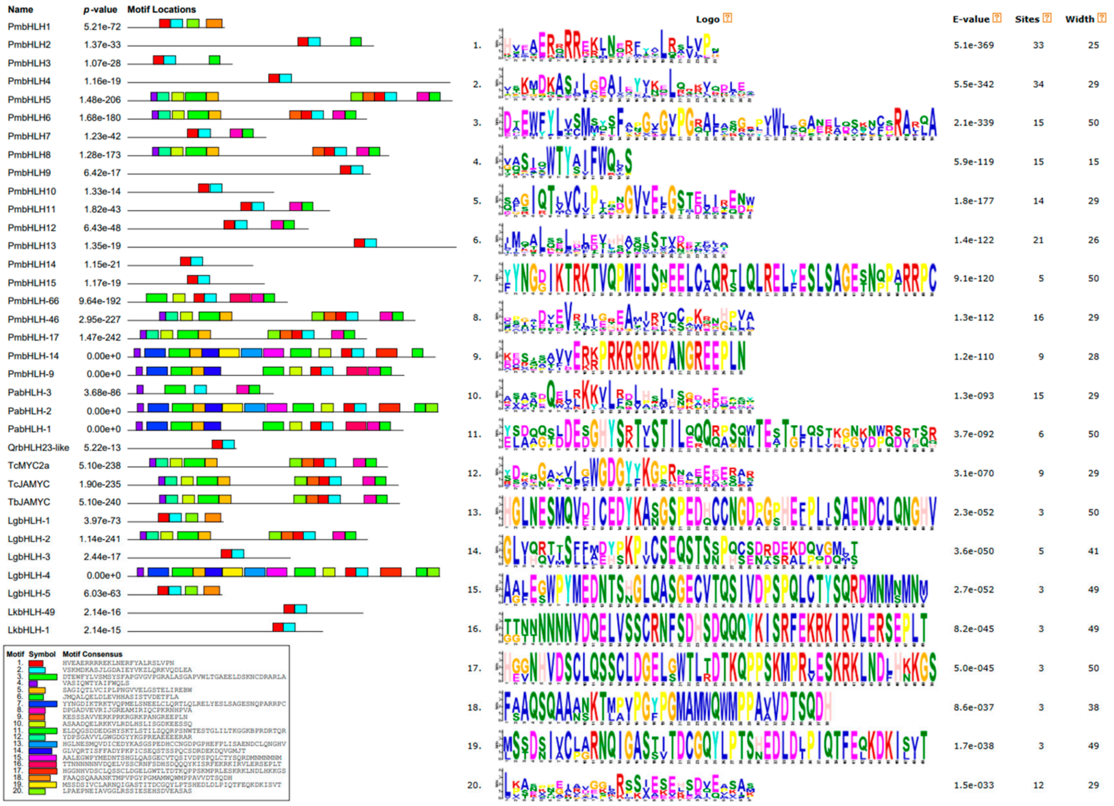

Figure S3. Motif analysis of conifer bHLH sequence combination downloaded by NCBI and PmbHLHs.

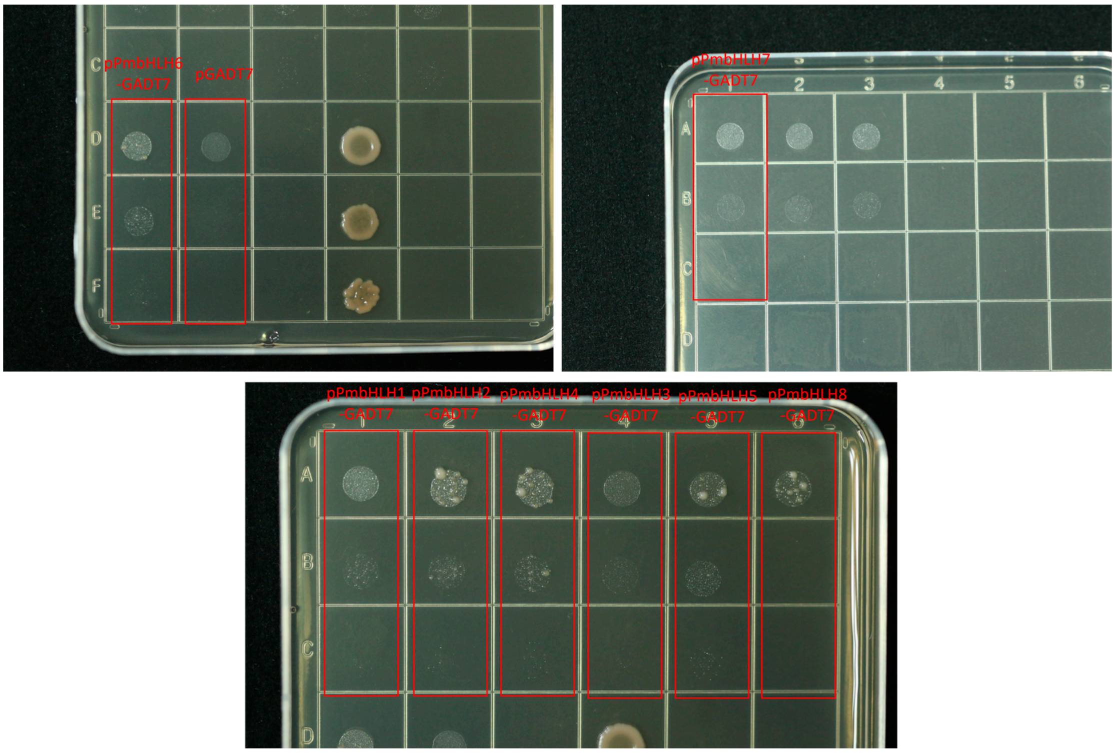

Figure S4. Yeast one-hybrid of PmbHLH1/2/3/4/5/6/7/8 with *Pm TPS (−)-α-pinene* promoter on SD/-Trp/-His/-Leu/140mM 3-AT plates, respectively.

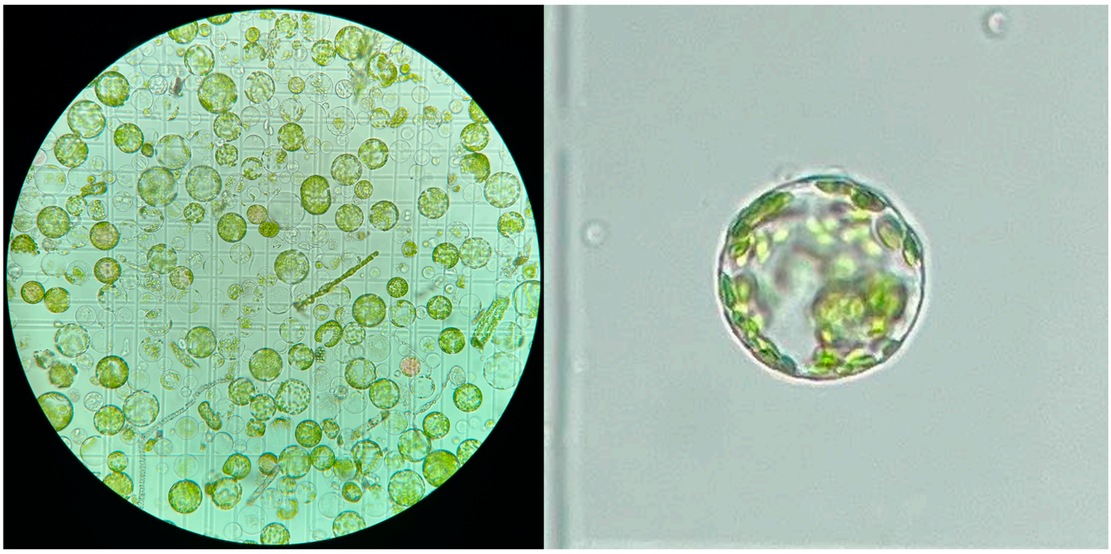

Figure S5. The protoplasts of *P. massoniana* were counted using hemocytometer.
