# Supplementary material for: Full-Length Transcriptome Sequencing of Pinus massoniana Under Simulated Monochamus alternatus Feeding Highlights bHLH Transcription Factor Involved in Defense Response
Source: Plants (Basel). 2025 Jul 3;14(13):2038. doi: 10.3390/plants14132038 (PMC12251683; doi:10.3390/plants14132038)
Supplement: Supplementary file 1 [file plants-14-02038-s001.zip › Table S1. Unigenes annotation result statistics.pdf]

Table S1. Unigenes annotation result statistics

Values	Total	Nr	Nt	Swissprot	KEGG	KOG	Pfam	GO	Intersection	Overall
Number	671,044	523,546	463,761	378,461	388,234	396,923	384,084	408,290	187,767	585,792
Percentage	100%	78.02%	69.11%	56.40%	57.86%	59.15%	57.24%	60.84%	27.98%	87.30%
