# Supplementary material for: Full-Length Transcriptome Sequencing of Pinus massoniana Under Simulated Monochamus alternatus Feeding Highlights bHLH Transcription Factor Involved in Defense Response
Source: Plants (Basel). 2025 Jul 3;14(13):2038. doi: 10.3390/plants14132038 (PMC12251683; doi:10.3390/plants14132038)
Supplement: Supplementary file 1 [file plants-14-02038-s001.zip › Table S2. Summary of DNBSEQ sequencing quality.pdf]

Table S2. Summary of DNBSEQ sequencing quality.

Sample Name	Total Raw Reads (M)	Total Clean Reads (M)	Total Clean Bases (Gb)	Clean Reads Q30(%)	Clean Reads Ratio (%)	Total Mapping (%)	Uniquely Mapping (%)
CK1	59.6	55.39	8.31	90.02	92.93	83.45	4.45
CK2	59.6	55.55	8.33	89.43	93.22	82.15	4.77
CK3	59.6	55.07	8.26	89.76	92.4	82.27	5.11
T1	59.6	55.24	8.29	89.77	92.68	82.84	4.39
T2	59.6	55.24	8.29	89.82	92.69	87.27	4.11
T3	59.6	55.1	8.27	90.16	92.46	84.39	4.69
