# Supplementary material for: Full-Length Transcriptome Sequencing of Pinus massoniana Under Simulated Monochamus alternatus Feeding Highlights bHLH Transcription Factor Involved in Defense Response
Source: Plants (Basel). 2025 Jul 3;14(13):2038. doi: 10.3390/plants14132038 (PMC12251683; doi:10.3390/plants14132038)
Supplement: Supplementary file 1 [file plants-14-02038-s001.zip › Table S3. KEGG Pathway Enrichment of DEGs in P. massoniana.pdf]

Table S3. KEGG Pathway Enrichment of DEGs in *P. massoniana*

Pathway ID	Pathway Name	Level 1	Level 2	Term Candidate Gene Num	Term Gene Num	*P* value
ko04626	Plant-pathogen interaction	Organismal Systems	Environmental adaptation	368	10418	$5.32E^{-18}$
ko04712	Circadian rhythm - plant	Organismal Systems	Environmental adaptation	88	2877	0.002168
ko00941	Flavonoid biosynthesis	Metabolism	Biosynthesis of other secondary metabolites	136	3479	$4.69E^{-10}$
ko00220	Arginine biosynthesis	Metabolism	Amino acid metabolism	59	1631	0.000263
ko00944	Flavone and flavonol biosynthesis	Metabolism	Biosynthesis of other secondary metabolites	39	1088	0.003021
ko00500	Starch and sucrose metabolism	Metabolism	Carbohydrate metabolism	182	6816	0.007568
ko00051	Fructose and mannose metabolism	Metabolism	Carbohydrate metabolism	83	2865	0.010499
ko00460	Cyanoamino acid metabolism	Metabolism	Metabolism of other amino acids	68	2314	0.01433
ko00591	Linoleic acid metabolism	Metabolism	Lipid metabolism	19	492	0.01575
ko00903	Limonene and pinene degradation	Metabolism	Metabolism of terpenoids and polyketides	24	656	0.013558
ko00910	Nitrogen metabolism	Metabolism	Energy metabolism	48	1533	0.013067
ko00943	Isoflavonoid biosynthesis	Metabolism	Biosynthesis of other secondary metabolites	10	203	0.016281
ko00900	Terpenoid backbone biosynthesis	Metabolism	Metabolism of terpenoids and polyketides	61	2170	0.040755
ko03018	RNA degradation	Genetic Information Processing	Folding, sorting and degradation	159	5484	0.000574
ko03013	RNA transport	Genetic Information Processing	Translation	373	14890	0.00978
ko04120	Ubiquitin mediated proteolysis	Genetic Information Processing	Folding, sorting and degradation	141	5328	0.021673

ko03022	Basal transcription factors	Genetic Information Processing	Transcription	49	1699	0.042906
ko02010	ABC transporters	Environmental Information Processing	Membrane transport	104	3096	$3.46E^{-05}$
ko04016	MAPK signaling pathway - plant	Environmental Information Processing	Signal transduction	231	8859	0.00812
