# Supplementary material for: Full-Length Transcriptome Sequencing of Pinus massoniana Under Simulated Monochamus alternatus Feeding Highlights bHLH Transcription Factor Involved in Defense Response
Source: Plants (Basel). 2025 Jul 3;14(13):2038. doi: 10.3390/plants14132038 (PMC12251683; doi:10.3390/plants14132038)
Supplement: Supplementary file 1 [file plants-14-02038-s001.zip › Table S4. Features of bHLH transcription factor DEG encoding sequences in P. massoniana.pdf]

Table S4. Features of *bHLH* transcription factor DEG encoding sequences in *P. massoniana*

Gene ID	Name	ORF (bp)	Amino acid	Molecular weight (kDa)	Hydrophobicity /hydrophilicity	Isoelectric point	Predicted subcellular localization
isoform_103739	*PmbHLH1*	714	237	26.38	-0.755	6.02	mito
isoform_527252	*PmbHLH2*	1803	600	64.23	-0.491	6.05	nucl
isoform_610753	*PmbHLH3*	771	256	28.87	-0.597	6.46	nucl
isoform_583229	*PmbHLH4*	2361	786	85.76	-0.812	6.29	nucl
isoform_2949	*PmbHLH5*	2376	791	87.15	-0.575	5.45	nucl
isoform_283768	*PmbHLH6*	1752	583	64.81	-0.572	7.25	nucl
isoform_599869	*PmbHLH7*	1167	388	41.55	-0.465	7.6	chlo
isoform_370450	*PmbHLH8*	1914	637	71.92	-0.631	7.68	nucl
isoform_14300	*PmbHLH9*	1776	592	64.52	-0.653	6.77	nucl
isoform_67352	*PmbHLH10*	1071	357	40	-0.374	8.7	nucl
isoform_56985	*PmbHLH11*	1479	493	54.99	-0.539	5.69	nucl
isoform_604410	*PmbHLH12*	1323	441	49.19	-0.52	5.58	nucl
isoform_665961	*PmbHLH13*	2406	801	84.88	-0.539	6.33	nucl
isoform_669985	*PmbHLH14*	918	306	33.23	-0.413	5.93	nucl
isoform_74091	*PmbHLH15*	1002	334	36.71	-0.561	9.14	nucl
