# Supplementary material for: Full-Length Transcriptome Sequencing of Pinus massoniana Under Simulated Monochamus alternatus Feeding Highlights bHLH Transcription Factor Involved in Defense Response
Source: Plants (Basel). 2025 Jul 3;14(13):2038. doi: 10.3390/plants14132038 (PMC12251683; doi:10.3390/plants14132038)
Supplement: Supplementary file 1 [file plants-14-02038-s001.zip › Table S5. Accession numbers of all AtbHLHs for phylogenetic tree.pdf]

Table S5. Accession numbers of all bHLHs for phylogenetic tree

Name	accession number	Name	accession number	Name	accession number	Name	accession number
AtbHLH1	AT5G51780.2	AtbHLH43	AT1G74500.1	AtbHLH85	AT1G25310.1	AtbHLH127	AT5G58010.1
AtbHLH2	AT4G16430.1	AtbHLH44	AT2G28160.1	AtbHLH86	AT1G12540.1	AtbHLH128	AT2G27230.1
AtbHLH3	AT4G00870.1	AtbHLH45	AT4G09180.1	AtbHLH87	AT2G31730.1	AtbHLH129	AT5G67060.1
AtbHLH4	AT1G01260.1	AtbHLH46	AT2G42280.3	AtbHLH88	AT2G14760.3	AtbHLH130	AT4G30980.2
AtbHLH5	AT5G54680.1	AtbHLH47	AT1G35460.1	AtbHLH89	AT2G22760.1	AtbHLH131	AT1G22380.1
AtbHLH6	AT2G43140.2	AtbHLH48	AT1G51140.1	AtbHLH90	AT2G22750.3	AtbHLH132	AT2G24260.1
AtbHLH7	AT3G56980.1	AtbHLH49	AT2G43060.1	AtbHLH91	AT2G40200.1	AtbHLH133	AT5G61270.1
AtbHLH8	AT3G56970.1	AtbHLH50	AT2G46510.1	AtbHLH92	AT3G21330.1	AtbHLH134	AT4G17880.1
AtbHLH9	AT2G31220.2	AtbHLH51	AT5G48560.1	AtbHLH93	AT3G19500.1	AtbHLH135	AT5G64340.1
AtbHLH10	AT2G31210.1	AtbHLH52	AT1G10120.1	AtbHLH94	AT3G22100.1	AtbHLH136	AT3G06120.1
AtbHLH11	AT3G25710.1	AtbHLH53	AT3G07340.1	AtbHLH95	AT3G23690.1	AtbHLH137	AT3G28910.1
AtbHLH12	AT1G06170.1	AtbHLH54	AT2G30766.1	AtbHLH96	AT3G56770.1	AtbHLH138	AT1G02340.1
AtbHLH13	AT3G23210.1	AtbHLH55	AT5G50010.1	AtbHLH97	AT2G31215.1	AtbHLH139	AT5G53210.1
AtbHLH14	AT1G72210.1	AtbHLH56	AT5G09460.1	AtbHLH98	AT1G62975.1	AtbHLH140	AT3G22275.1
AtbHLH15	AT1G22490.2	AtbHLH57	AT4G38070.1	AtbHLH99	AT1G05805.1	AtbHLH141	AT2G43010.5
AtbHLH16	AT2G41130.1	AtbHLH58	AT1G29270.1	AtbHLH100	AT1G10585.1	AtbHLH142	AT2G47270.1
AtbHLH17	AT1G51070.2	AtbHLH59	AT4G05170.1	AtbHLH101	AT3G28857.1	AtbHLH143	AT4G21330.1
AtbHLH18	AT5G15160.1	AtbHLH60	AT4G01460.1	AtbHLH102	AT5G09750.1	AtbHLH144	AT1G63650.1
AtbHLH19	AT5G43650.1	AtbHLH61	AT4G28800.2	AtbHLH103	AT5G39240.1	AtbHLH145	AT3G26744.1
AtbHLH20	AT5G65640.1	AtbHLH62	AT4G29930.3	AtbHLH104	AT5G57780.1	AtbHLH146	AT2G20180.2
AtbHLH21	AT2G42300.1	AtbHLH63	AT4G20970.1	AtbHLH105	AT2G18969.1	PmbHLH 66	UGN74588.1
AtbHLH22	AT3G57800.1	AtbHLH64	AT4G37850.1	AtbHLH106	AT3G29370.1	PmbHLH 51	UGN74587.1
AtbHLH23	AT1G71200.4	AtbHLH65	AT4G25400.2	AtbHLH107	AT3G47640.1	PmbHLH 46	UGN74586.1
AtbHLH24	AT3G47710.1	AtbHLH66	AT4G25410.1	AtbHLH108	AT5G01305.1	PmbHLH 44	UGN74585.1
AtbHLH25	AT1G68920.1	AtbHLH67	AT4G21340.1	AtbHLH109	AT3G50330.1	PmbHLH 17	UGN74584.1
AtbHLH26	AT4G36060.3	AtbHLH68	AT5G51790.3	AtbHLH110	AT4G36930.1	PmbHLH 14	UGN74583.1
AtbHLH27	AT3G19860.2	AtbHLH69	AT5G65320.1	AtbHLH111	AT5G67110.1	PmbHLH 9	UGN74582.1
AtbHLH28	AT5G50915.1	AtbHLH70	AT5G62610.1	AtbHLH112	AT4G14080.1	PabHLH-3	ANB66422.1
AtbHLH29	AT5G56960.2	AtbHLH71	AT5G57150.4	AtbHLH113	AT1G26945.1	PabHLH-2	ANB66421.1
AtbHLH30	AT4G28790.1	AtbHLH72	AT1G09250.1	AtbHLH114	AT1G75910.1	PabHLH-1	ANB66420.1
AtbHLH31	AT5G39860.1	AtbHLH73	AT1G29950.1	AtbHLH115	AT2G46970.1	QrbHLH23-like	XP_050266471.1
AtbHLH32	AT3G59060.2	AtbHLH74	AT1G68240.1	AtbHLH116	AT3G06590.1	TcMYC2a	ATY38591.1
AtbHLH33	AT5G04150.1	AtbHLH75	AT1G27660.1	AtbHLH117	AT3G62090.2	TcJAMYC	ACM48567.1
AtbHLH34	AT5G46690.1	AtbHLH76	AT4G28811.1	AtbHLH118	AT1G27740.1	TbJAMYC	APP91306.1
AtbHLH35	AT4G14410.1	AtbHLH77	AT4G28815.1	AtbHLH119	AT2G18300.3	CjbHLH	XP_057869179.2
AtbHLH36	AT2G41240.1	AtbHLH78	AT1G10586.1	AtbHLH120	AT3G28470.1	LgbHLH 1	UNP37118.1
AtbHLH37	AT4G09820.1	AtbHLH79	AT4G38062.1	AtbHLH121	AT1G64625.1	LgbHLH 2	UNP37117.1
AtbHLH38	AT1G03040.3	AtbHLH80	AT4G38065.1	AtbHLH122	AT1G56100.1	LgbHLH 3	UNP37116.1
AtbHLH39	AT5G46830.1	AtbHLH81	AT1G49830.1	AtbHLH123	AT5G21040.1	LgbHLH 4	UNP37115.1
AtbHLH40	AT4G29100.1	AtbHLH82	AT1G68810.1	AtbHLH124	AT3G15400.1	LgbHLH 5	UNP37114.1
AtbHLH41	AT4G02590.1	AtbHLH83	AT1G10610.1	AtbHLH125	AT5G08130.8	LkbHLH 49	QZZ92769.1
AtbHLH42	AT3G02150.2	AtbHLH84	AT1G05710.10	AtbHLH126	AT1G12860.1	LkbHLH 1	WBK62437.1
