# Supplementary material for: Full-Length Transcriptome Sequencing of Pinus massoniana Under Simulated Monochamus alternatus Feeding Highlights bHLH Transcription Factor Involved in Defense Response
Source: Plants (Basel). 2025 Jul 3;14(13):2038. doi: 10.3390/plants14132038 (PMC12251683; doi:10.3390/plants14132038)
Supplement: Supplementary file 1 [file plants-14-02038-s001.zip › Table S6. qPCR primers of PmbHLHs.pdf]

Table S6. qPCR primers of *PmbHLHs*

Gene name	Primer	Primer sequence (5′-3′)	Efficiency (%)	(R^2)
PmbHLH1	QbHLH1-F	ATTCTTCTGGTGCGGTCAGG	99.98	0.992
	QbHLH1-R	CTCACGGCAAGCTTTTGACC		
PmbHLH2	QbHLH2-F	TGCCAACGTTCAAGGCAATG	96.87	0.9783
	QbHLH2-R	ACAGCATATTGGGAGGCTCG		
PmbHLH3	QbHLH3-F	TGAAATTTGCAACGCAGGGG	98.38	0.981
	QbHLH3-R	CACGTTGACAGTGAGGGAGT		
PmbHLH4	QbHLH4-F	GCCGTGTTCGATCCTCAGAT	100.20	0.995
	QbHLH4-R	TTGGGGAAGCTCCATGCAAT		
PmbHLH5	QbHLH5-F	CCCGTTTTCCGTCCACCTTA	101.23	0.9926
	QbHLH5-R	GCAACACCAAGTGGCGAAAT		
PmbHLH6	QbHLH6-F	CCCGTTTTCCGTCCACCTTA	104.12	0.9997
	QbHLH6-R	GCAACACCAAGTGGCGAAAT		
PmbHLH7	QbHLH7-F	GGAAAGCCATCCCGTTGGTA	97.23	0.999
	QbHLH7-R	TTAATGTCTGGGTTCCGCCC		
PmbHLH8	QbHLH8-F	TCCCATGCACCATCTGTAGC	102.3	0.999
	QbHLH8-R	TGCAACTGGATGACACTCCC		
UBE2D	QUBE-F	GTCCGTAATTCGCATCATAG	99.66	0.989
	QUBE-R	CAGCTTACAGAACAAACCCTA		
